# A Comparative Study for Flow of Viscoelastic Fluids with Cattaneo-Christov Heat Flux

**DOI:** 10.1371/journal.pone.0155185

**Published:** 2016-05-13

**Authors:** Tasawar Hayat, Taseer Muhammad, Ahmed Alsaedi, Meraj Mustafa

**Affiliations:** 1 Department of Mathematics, Quaid-I-Azam University 45320, Islamabad 44000, Pakistan; 2 Nonlinear Analysis and Applied Mathematics (NAAM) Research Group, Faculty of Science, King Abdulaziz University, P. O. Box 80203, Jeddah 21589, Saudi Arabia; 3 School of Natural Sciences (SNS), National University of Sciences and Technology (NUST), Islamabad 44000, Pakistan; Tianjin University, CHINA

## Abstract

This article examines the impact of Cattaneo-Christov heat flux in flows of viscoelastic fluids. Flow is generated by a linear stretching sheet. Influence of thermal relaxation time in the considered heat flux is seen. Mathematical formulation is presented for the boundary layer approach. Suitable transformations lead to a nonlinear differential system. Convergent series solutions of velocity and temperature are achieved. Impacts of various influential parameters on the velocity and temperature are sketched and discussed. Numerical computations are also performed for the skin friction coefficient and heat transfer rate. Our findings reveal that the temperature profile has an inverse relationship with the thermal relaxation parameter and the Prandtl number. Further the temperature profile and thermal boundary layer thickness are lower for Cattaneo-Christov heat flux model in comparison to the classical Fourier’s law of heat conduction.

## Introduction

Mechanism of heat transfer occurs when there is a difference of temperature between the bodies or between the various parts of the same body. Such mechanism has widespread industrial and technological applications like cooling of nuclear reactors, cooling of electronic devices, energy production, power generation and many others [1–3]. Fourier [4] was the first who developed the classical law of heat conduction. This well known law has been the basis to study the heat transfer mechanism since it appeared in the literature. But one of the major limitation of this model is that it leads to a parabolic energy equation which means that an initial disturbance would instantly experienced by the system under consideration. This fact is referred in literature as “Paradox of heat conduction”. To overcome this limitation, Cattaneo [5] modified this law by adding a relaxation time term. Then Christov [6] further modified the Cattaneo model [5] by replacing the ordinary derivative with the Oldroyd's upper-convected derivative in order to maintain the frame-indifferent generalization. He developed a single energy equation for the governing problem. This model is known as the Cattaneo-Christov heat flux model. Ciarletta and Straughan [7] explored the uniqueness and structural stability of solutions for energy equation using Cattaneo-Christov theory. Straughan [8] used the Cattaneo-Christov heat flux model for the thermal convection in a horizontal layer of viscous fluid. Straughan [9, 10] also examined the models of acoustic waves and Gene-culture shock waves using the heat flux by Cattaneo-Christov theory. Han et al. [11] analyzed the flow of Maxwell liquid past a linearly stretching surface through the Cattaneo-Christov heat flux model. Mustafa [12] employed the Cattaneo-Christov theory in stretched flow and heat transfer of Maxwell liquid. He developed both analytic and numeric solutions of the governing problems. Recently Khan et al. [13] performed a numerical study to examine the thermal relaxation in the flow of Maxwell liquid by an exponentially stretching surface.

The analysis of boundary-layer flow past a stretching surface is significant in various industrial and technological processes. Examples of such practical applications are wire drawing, extrusion of plastic sheets, hot rolling, paper production, glass fiber etc. There are many fluids in our daily life usage like shampoos, certain oils, sugar solution, tomato paste, mud, apple sauce, chyme, personal care products and several others which do not satisfy the classical Newton's law of viscosity. Such fluids fall in the class of non-Newtonian fluids. All the non-Newtonian fluids through their distinct features cannot be explained by using a single constitutive relationship. This fact of non-Newtonian fluids are quite distinct than that of viscous fluids. In the past various models have been developed to characterize the properties of non-Newtonian fluids. Amongst these the simplest subclasses of differential type fluids are the elastico-viscous and second grade [14–20]. Further the study of gas-liquid two-phase flow is widely encountered in several industrial processes like natural gas networks, lubrication, spray processes, nuclear reactor cooling etc. Thus Gao et al. [21] performed a multivariate weighted complex network analysis to explore the nonlinear dynamic behavior in two-phase flow. Gao et al. [22] also reported the multi-frequency complex network to study the uncovering oil-water flow structure. Recently Gao et al. [23] examined the slug to churn flow transition by considering the multivariate pseudo Wigner distribution and multivariate multiscale entropy.

This communication presents a comparative study for Cattaneo-Christov heat flux model in boundary layer flow by considering the two classes of viscoelastic fluids. Constitutive relations for second grade and elastico-viscous fluids are considered. Most of the studies in the literature are explained through the classical Fourier’s law of heat conduction. There is not a single study in the literature that present the characteristics of Cattaneo-Christov heat flux in boundary layer flow of viscoelastic fluids. Hence the purpose here is to employ the Cattaneo-Christov heat flux model in the boundary layer flow of viscoelastic fluids. To the best of the author's knowledge, no such consideration has been discussed in the literature yet. Similarity approach is adopted to convert the partial differential system into the set of nonlinear ordinary differential system. The governing nonlinear system is solved through the homotopy analysis method (HAM) [24–32]. Impacts of various influential parameters on the velocity and temperature are studied and examined.

## Formulation

We consider the steady two-dimensional (2D) flows of viscoelastic fluids over a linear stretching surface. Flow models for second grade and elastico-viscous fluids are considered. The Cartesian coordinate system is adopted in such a way that the *x*—axis is taken along the stretching surface and *y*—axis is orthogonal to it. Let *U*_*w*_(*x*) = *ax* denotes the surface stretching velocity along the *x*—direction. The heat transfer process is studied through the Cattaneo-Christov heat flux theory. The governing two-dimensional (2D) boundary-layer flows in two cases of fluids are under consideration

$$\frac{\partial u}{\partial x}+\frac{\partial v}{\partial y}=0,$$ (1)

$$u\frac{\partial u}{\partial x}+v\frac{\partial u}{\partial y}=\nu \frac{\partial^{2}u}{\partial y^{2}}-k_{0}\left(u\frac{\partial^{3}u}{\partial x\partial y^{2}}+v\frac{\partial^{3}u}{\partial y^{3}}-\frac{\partial u}{\partial y}\frac{\partial^{2}u}{\partial x\partial y}+\frac{\partial u}{\partial x}\frac{\partial^{2}u}{\partial y^{2}}\right),$$ (2)

$$\rho c_{p}\left(u\frac{\partial T}{\partial x}+v\frac{\partial T}{\partial y}\right)=-\nabla .q.$$ (3)

Note that (*u*,*v*) are the fluid velocities in horizontal and vertical directions respectively, *v* = *μ* / *ρ* the kinematic viscosity, *μ* the dynamic viscosity, *ρ* the density, *c*_*p*_ the specific heat, *k*_0_ = −*α*_1_ / *ρ* the elastic parameter, *T* the temperature and **q** the heat flux. Here *k*_0_ > 0 is for elastico-viscous fluid, *k*_0_ < 0 corresponds to a second grade fluid and *k*_0_ = 0 is for Newtonian fluid. According to the Cattaneo-Christov heat flux model [3], one can write

$$q+\lambda \left(\frac{\partial q}{\partial t}+V.\nabla q-q.\nabla V+\left(\nabla .V\right)\;q\right)=-k\nabla T,$$ (4)

where *λ* is the relaxation time of heat flux and *k* the thermal conductivity. For *λ* = 0, Eq (4) is reduced to the classical Fourier's law of heat conduction. Omitting **q** from Eqs (3) and (4), the energy equation becomes

$$u\frac{\partial T}{\partial x}+v\frac{\partial T}{\partial y}+\lambda \left(\begin{array}{c} u\frac{\partial u}{\partial x}\frac{\partial T}{\partial x}+v\frac{\partial v}{\partial y}\frac{\partial T}{\partial y}+u\frac{\partial v}{\partial x}\frac{\partial T}{\partial y}\\ +v\frac{\partial u}{\partial y}\frac{\partial T}{\partial x}+2uv\frac{\partial^{2}T}{\partial x\partial y}+u^{2}\frac{\partial^{2}T}{\partial x^{2}}+v^{2}\frac{\partial^{2}T}{\partial y^{2}} \end{array}\right)=\alpha \frac{\partial^{2}T}{\partial y^{2}}.$$ (5)

The subjected boundary conditions are

$$u=U_{w}\left(x\right)=ax,\;v=0,\;T=T_{w}\text{ at }y=0,$$ (6)

$$u\to 0,\;T\to T_{\infty }\text{ as }y\to \infty ,$$ (7)

in which *α* = *k* / *ρc*_*p*_ is the thermal diffusivity, *T*_*w*_ the constant surface temperature, *T*_∞_ the ambient fluid temperature and *a* the positive constant. Using

$$\begin{array}{c} u=axf^{\prime }(\eta ),\;v=-{\left(a\nu \right)}^{1/2}f(\eta ),\\ \theta (\eta )=\left(T-T_{\infty }\right)/\left(T_{w}-T_{\infty }\right),\;\eta ={\left(\frac{a}{\nu }\right)}^{1/2}y. \end{array}$$ (8)

Now Eq (1) is satisfied and Eqs (2) and (5)–(7) lead to the following forms

$$f^{\prime \prime \prime }+ff^{\prime \prime }-{\left(f^{\prime }\right)}^{2}-k_{1}^{*}\left(2f^{\prime }f^{\prime \prime \prime }-{\left(f^{\prime \prime }\right)}^{2}-ff^{iv}\right)=0,$$ (9)

$$\frac{1}{\text{Pr}}\theta^{\prime \prime }+f\theta^{\prime }-\gamma \left(ff^{\prime }\theta^{\prime }+f^{2}\theta^{\prime \prime }\right)=0,$$ (10)

$$f=0,\;f^{\prime }=1,\;\theta =1\text{ at }\eta =0,$$ (11)

$$f^{\prime }\to 0,\;\theta \to 0\text{ as }\eta \to \infty .$$ (12)

In the above expressions $k_{1}^{*}$ is the viscoelastic parameter, Pr the Prandtl number and *γ* the thermal relaxation parameter. It is noticed that $k_{1}^{*}>0$ for elastico-viscous fluid and $k_{1}^{*}<0$ for the second grade fluid. These parameters have values

$$k_{1}^{*}=-\frac{k_{0}a}{\nu },\text{ Pr}=\frac{\nu }{\alpha },\;\gamma =\lambda a.$$ (13)

The expression of the skin friction coefficient is

$$C_{f}=\frac{{\tau_{w}|}_{y=0}}{\rho U_{w}^{2}}=\frac{{\left(\nu \frac{\partial u}{\partial y}-k_{0}\left(u\frac{\partial^{2}u}{\partial x\partial y}-2\frac{\partial u}{\partial y}\frac{\partial v}{\partial y}+v\frac{\partial^{2}u}{\partial y^{2}}\right)\right)}_{y=0}}{U_{w}^{2}}.$$ (14)

Skin friction coefficient through dimensionless scale is

$$\text{R}{\text{e}}_{x}^{1/2}C_{f}=\left(1-3k_{1}^{*}\right)\;f^{\prime \prime }(0),$$ (15)

in which Re_*x*_ = *U*_*w*_*x*/*v* represents the local Reynolds number.

## Homotopic Solutions

The appropriate initial approximations (*f*_0_,*θ*_0_), linear operators (**L**_*f*_,**L**_*θ*_) and deformation problems at zeroth and *m* th orders are

$$f_{0}(\eta )=1-\text{exp}\left(-\eta \right),\;\theta_{0}(\eta )=\text{exp}\left(-\eta \right),$$ (16)

$$L_{f}=\frac{d^{3}f}{d\eta^{3}}-\frac{df}{d\eta },\;L_{\theta }=\frac{d^{2}\theta }{d\eta^{2}}-\theta ,$$ (17)

$$L_{f}\left[B_{1}+B_{2}\text{exp}\left(\eta \right)+B_{3}\text{exp}\left(-\eta \right)\right]=0,\;L_{\theta }\left[B_{4}\text{exp}\left(\eta \right)+B_{5}\text{exp}\left(-\eta \right)\right]=0,$$ (18)

$$(1-\text{Þ})L_{f}\left[\hat{f}(\eta ,\text{ Þ})-f_{0}(\eta )\right]=\text{Þ}\hbar_{f}N_{f}[\hat{f}(\eta ,\text{ Þ})],$$ (19)

$$(1-\text{Þ})L_{\theta }\left[\hat{\theta }(\eta ,\text{ Þ})-\theta_{0}(\eta )\right]=\text{Þ}\hbar_{\theta }N_{\theta }[\hat{f}(\eta ,\text{ Þ}),\;\hat{\theta }(\eta ,\text{ Þ})],$$ (20)

$$\hat{f}(0,\text{ Þ})=0,\;{\hat{f}}^{\prime }(0,\text{ Þ})=1,\;{\hat{f}}^{\prime }(\infty ,\text{ Þ})=0,\;\hat{\theta }(0,\text{ Þ})=1,\;\hat{\theta }(\infty ,\text{ Þ})=0,$$ (21)

$$N_{f}\left[\hat{f}(\eta ;\text{ Þ})\right]=\frac{\partial^{3}\hat{f}}{\partial \eta^{3}}+\hat{f}\frac{\partial^{2}\hat{f}}{\partial \eta^{2}}-{\left(\frac{\partial \hat{f}}{\partial \eta }\right)}^{2}-k_{1}^{*}\left(2\frac{\partial \hat{f}}{\partial \eta }\frac{\partial^{3}\hat{f}}{\partial \eta^{3}}-{\left(\frac{\partial^{2}\hat{f}}{\partial \eta^{2}}\right)}^{2}-\hat{f}\frac{\partial^{4}\hat{f}}{\partial \eta^{4}}\right),$$ (22)

$$N_{\theta }\left[\hat{f}(\eta ;\text{ Þ}),\;\hat{\theta }(\eta ,\text{ Þ})\right]=\frac{1}{\text{Pr}}\frac{\partial^{2}\hat{\theta }}{\partial \eta^{2}}+\hat{f}\frac{\partial \hat{\theta }}{\partial \eta }-\gamma \left(\hat{f}\frac{\partial \hat{f}}{\partial \eta }\frac{\partial \hat{\theta }}{\partial \eta }+{\hat{f}}^{2}\frac{\partial^{2}\hat{\theta }}{\partial \eta^{2}}\right),$$ (23)

$$L_{f}\left[f_{m}(\eta )-\chi_{m}f_{m-1}(\eta )\right]=\hbar_{f}R_{f}^{m}(\eta ),$$ (24)

$$L_{\theta }\left[\theta_{m}(\eta )-\chi_{m}\theta_{m-1}(\eta )\right]=\hbar_{\theta }R_{\theta }^{m}(\eta ),$$ (25)

$$f_{m}(0)=f_{m}^{\prime }(0)=f_{m}^{\prime }(\infty )=0,\;\theta_{m}(0)=\theta_{m}(\infty )=0,$$ (26)

$$R_{f}^{m}(\eta )=f_{m-1}^{\prime \prime \prime }+\overset{m-1}{\underset{k=0}{\sum }}\left\{f_{m-1-k}f_{k}^{\prime \prime }-f_{m-1-k}^{\prime }f_{k}^{\prime }-2k_{1}^{*}f_{m-1-k}^{\prime }f_{k}^{\prime \prime \prime }+k_{1}^{*}f_{m-1-k}^{\prime \prime }f_{k}^{\prime \prime }+k_{1}^{*}f_{m-1-k}f_{k}^{iv}\right\},$$ (27)

$$R_{\theta }^{m}(\eta )=\frac{1}{\text{Pr}}\theta_{m-1}^{\prime \prime }+\overset{m-1}{\underset{k=0}{\sum }}f_{m-1-k}\theta_{k}^{\prime }-\gamma \overset{m-1}{\underset{k=0}{\sum }}f_{m-1-k}\overset{k}{\underset{l=0}{\sum }}f_{k-l}^{\prime }\theta_{l}^{\prime }-\gamma \overset{m-1}{\underset{k=0}{\sum }}f_{m-1-k}\overset{k}{\underset{l=0}{\sum }}f_{k-l}\theta_{l}^{\prime \prime },$$ (28)

$$\chi_{m}=\{\begin{array}{c} 0,\;m\leq 1,\\ 1,\;m>1. \end{array}$$ (29)

Here Þ∈[0,1] is the embedding parameter, ℏ_*f*_ and ℏ_*θ*_ the non-zero auxiliary parameters and **N**_*f*_ and **N**_*θ*_ the nonlinear operators. The expressions of general solutions (*f*_*m*_,*θ*_*m*_) of the Eqs (24) and (25) through the special solutions $\left(f_{m}^{*},\;\theta_{m}^{*}\right)$ are presented as follows:

$$f_{m}(\eta )=f_{m}^{*}(\eta )+B_{1}+B_{2}\text{exp}\left(\eta \right)+B_{3}\text{exp}\left(-\eta \right),$$ (30)

$$\theta_{m}(\eta )=\theta_{m}^{*}(\eta )+B_{4}\text{exp}\left(\eta \right)+B_{5}\text{exp}\left(-\eta \right),$$ (31)

in which the constants *B*_*j*_ (*j* = 1−5) subject to the boundary conditions Eq (26) are defined by

$$B_{2}=B_{4}=0,\;B_{3}={\frac{\partial f_{m}^{*}\left(\eta \right)}{\partial \eta }|}_{\eta =0},\;B_{1}=-B_{3}-f_{m}^{*}\left(0\right),\;B_{5}=-\theta_{m}^{*}(0).$$ (32)

## Convergence Analysis

**Fig 1:**
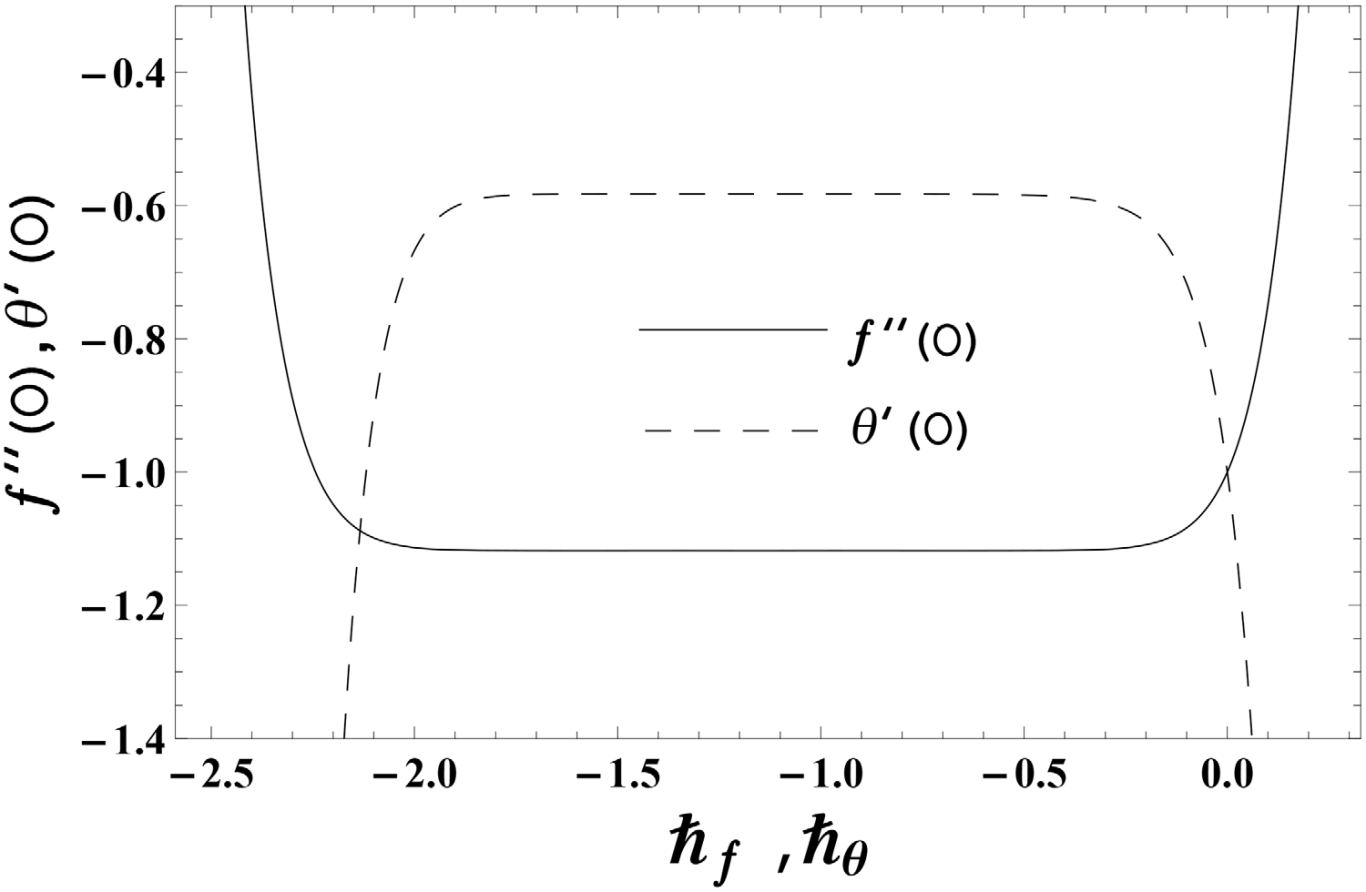
The ℏ—curves for *f*(*η)* and *θ*(*η*) in elastico-viscous fluid when $k_{1}^{*}=0.2,$ *γ* = 0.3 and Pr = 1.0.

**Fig 2:**
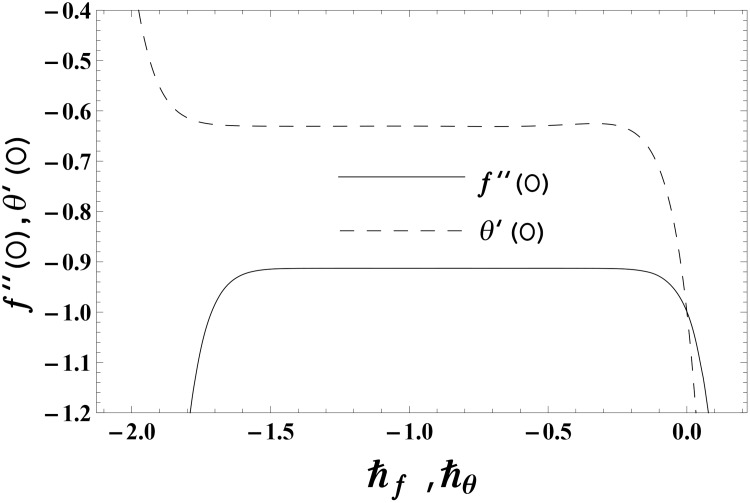
The ℏ—curves for *f*(*η)* and *θ*(*η*) in second grade fluid when $k_{1}^{*}=-0.2,$ *γ* = 0.3 and Pr = 1.0.

No doubt the approximate homotopic solutions contain the non-zero auxiliary parameters ℏ_*f*_ and ℏ_*θ*_. Such non-zero auxiliary parameters are important in accelerating the convergence of obtained homotopic solutions. The proper values of such parameters are important to get the convergent approximate homotopic solutions. To get the appropriate values of ℏ_*f*_ and ℏ_*θ*_, the ℏ—curves are plotted at 14th order of homotopic deformations. Figs 1 and 2 clearly indicate that the convergence region exists inside the ranges −1.80 ≤ ℏ_*f*_ ≤ −0.40 and −1.70 ≤ ℏ_*θ*_ ≤ −0.40 for elastico-viscous fluid $\left(k_{1}^{*}>0\right)$ and −1.50 ≤ ℏ_*f*_ ≤ −0.20 and −1.50 ≤ ℏ_*θ*_ ≤ −0.50 for second grade fluid $\left(k_{1}^{*}<0\right).$ Table 1 presents that 15th order of homotopic deformations is necessary for convergent approximate homotopic solutions in elastico-viscous fluid case whereas the 20th order of homotopic deformations is necessary for convergent approximate homotopic solutions in second grade fluid case (see Table 2).

**Table 1:** Convergence of homotopic solutions in elastico-viscous fluid for various order of homotopic approximations when $k_{1}^{*}=-0.2,$ *γ* = 0.3 and Pr = 1.0.

Order of approximations	−*f′′*(0)	−*θ′*(0)
1	1.10000	0.66667
5	1.11802	0.58484
10	1.11803	0.58279
15	1.11803	0.58273
25	1.11803	0.58273
35	1.11803	0.58273
50	1.11803	0.58273

**Table 2:** Convergence of homotopic solutions in second grade fluid for various order of homotopic approximations when $k_{1}^{*}=-0.2,$ *γ* = 0.3 and Pr = 1.0.

*Order of approximations*	−*f′′*(0)	−*θ′*(0)
1	0.90000	0.66667
5	0.91286	0.62357
10	0.91287	0.63155
15	0.91287	0.63028
20	0.91287	0.63052
25	0.91287	0.63052
35	0.91287	0.63052
50	0.91287	0.63052

## Discussion

**Fig 3:**
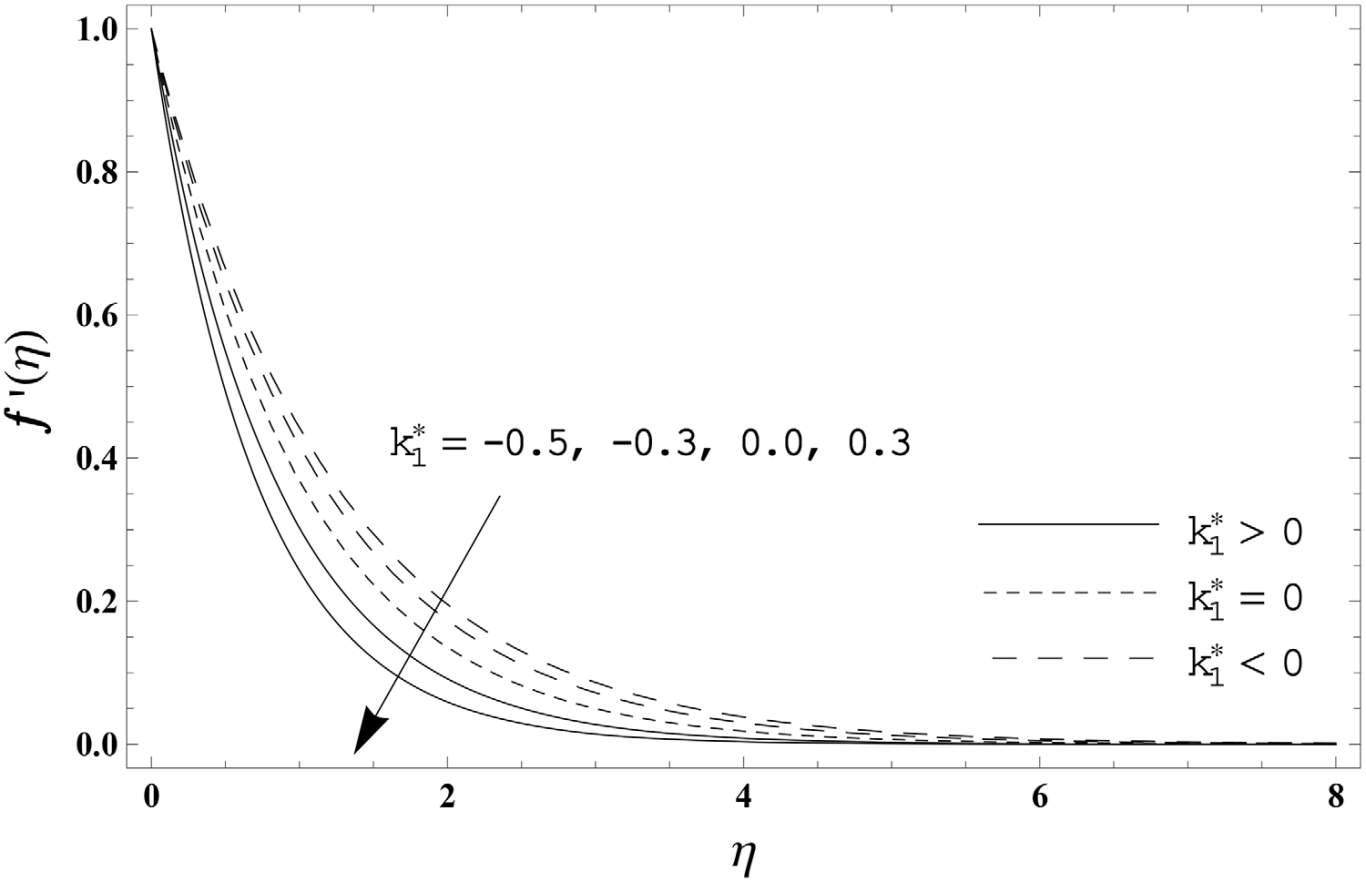
Effect of $k_{1}^{*}$ on *f′*(*η)*.

**Fig 4:**
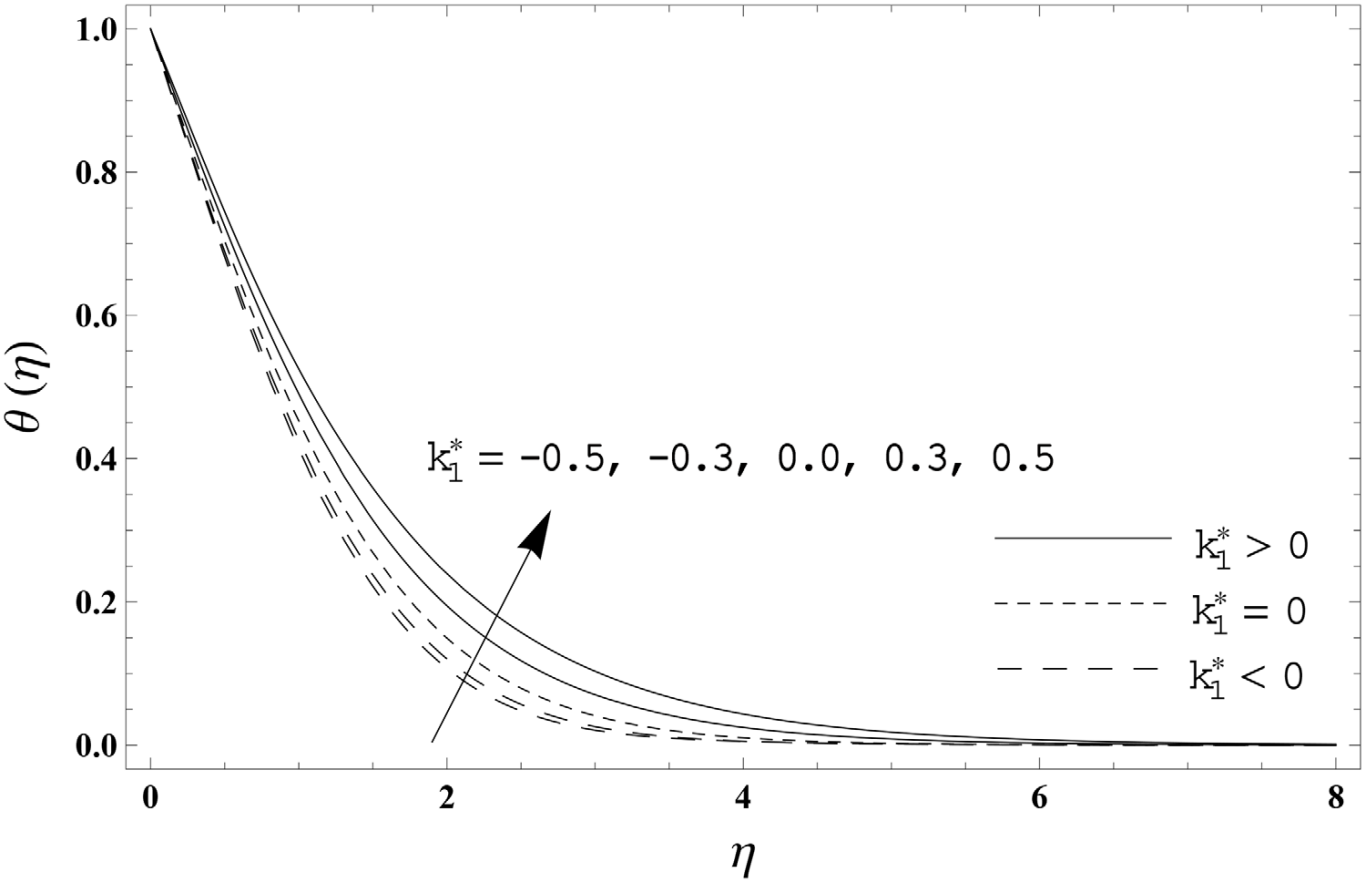
Effect of $k_{1}^{*}$ on *θ*(*η)* when *γ* = 0.3 and Pr = 1.0.

**Fig 5:**
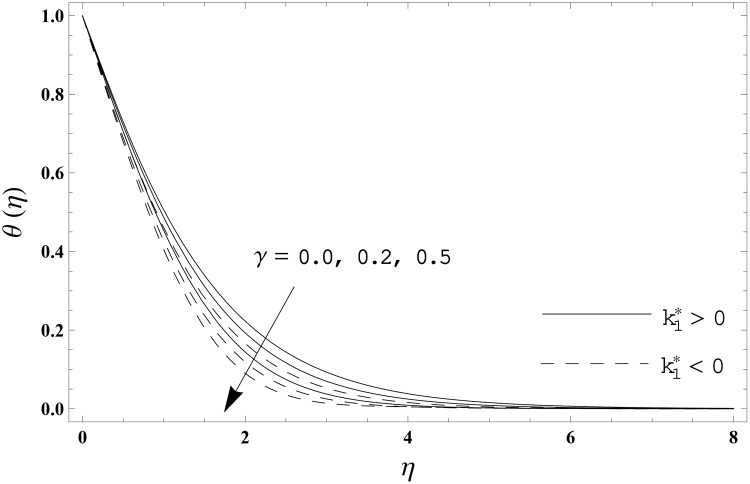
Effect of *γ* on *θ*(*η)* when Pr = 1.0.

**Fig 6:**
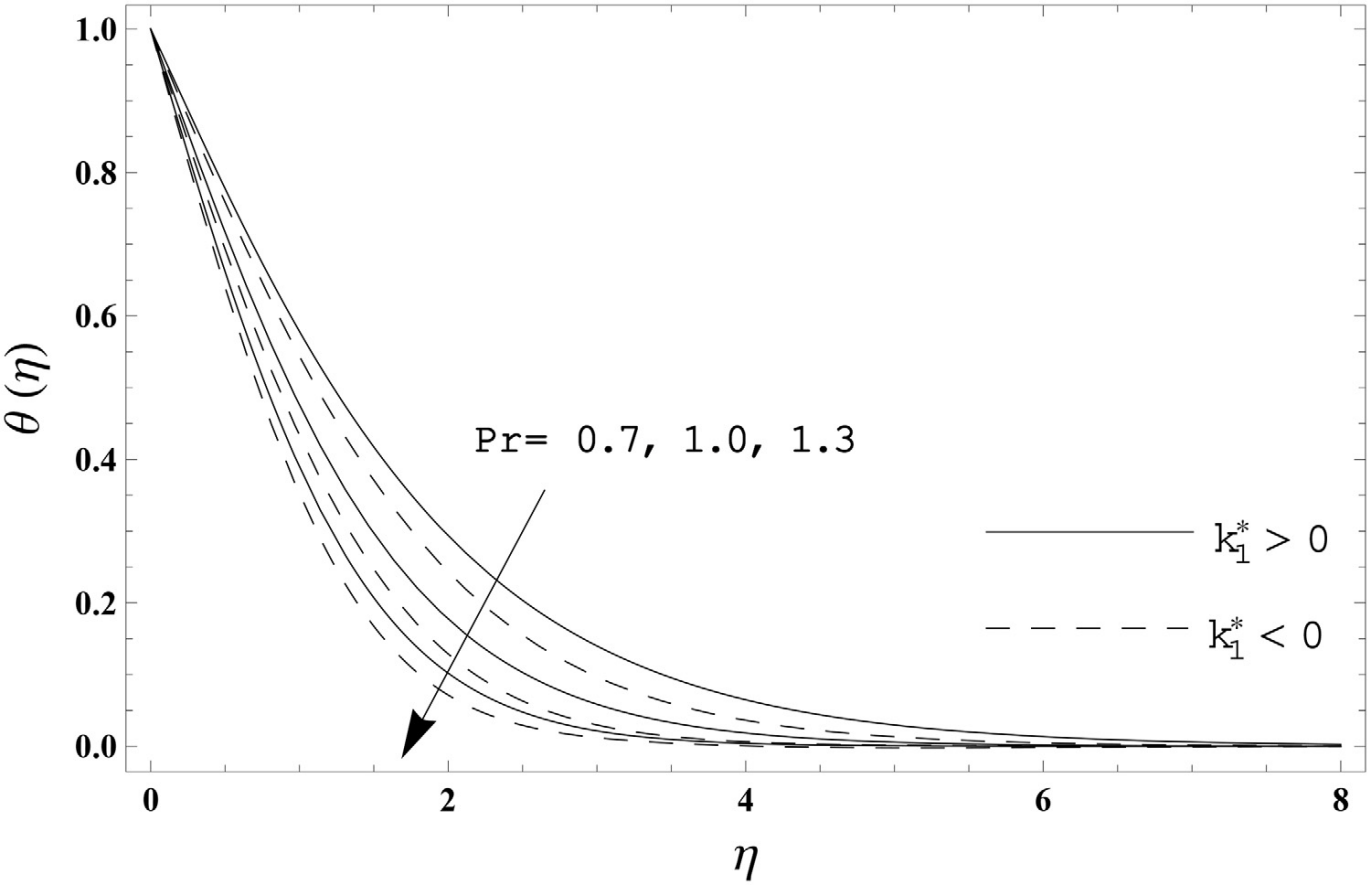
Effect of Pr on *θ*(*η)* when *γ* = 0.3.

The present section has been arranged to examine the impacts of viscoelastic parameter $k_{1}^{*}$, thermal relaxation parameter *γ* and Prandtl number Pr on the non-dimensional velocity distribution *f′*(*η)* and temperature distribution *θ*(*η)*. Here the elastico-viscous $\left(k_{1}^{*}>0\right)$ and second grade $\left(k_{1}^{*}<0\right)$ fluids are considered. This purpose is achieved through the plots 3–6. Impact of viscoelastic parameter $k_{1}^{*}$ on the velocity distribution *f′*(*η)* for both fluids is sketched in Fig 3. Here the velocity *f′*(*η)* is reduced for larger values of elastico-viscous parameter $\left(\;k_{1}^{*}>0\right)$ while the velocity *f′*(*η)* is higher for larger values of second grade parameter $\left(\;k_{1}^{*}<0\right)$. The results corresponds to the Newtonian fluid situation when $k_{1}^{*}=0$. Fig 4 presents the influence of viscoelastic parameter $k_{1}^{*}$ on the temperature distribution *θ*(*η)* for both fluids. Temperature distribution *θ*(*η)* and thermal boundary layer thickness are enhanced for elastico-viscous fluid while opposite behavior is observed in case of second grade fluid. Fig 5 presents the impact of thermal relaxation parameter *γ* on the temperature distribution *θ*(*η)*. Temperature distribution *θ*(*η)* and thermal boundary layer thickness are decreasing functions of thermal relaxation parameter. Here the thermal relaxation parameter *γ* = 0 shows that the heat flux expression is reduced to the classical Fourier's law. Effect of Prandtl number Pr on the temperature distribution *θ*(*η)* for both fluids is displayed in Fig 6. Both the temperature distribution *θ*(*η)* and thermal boundary layer thickness are reduced when Prandtl number Pr increases. Prandtl number has an inverse relationship with the thermal diffusivity. High Prandtl fluid possess weaker thermal diffusivity and the low Prandtl fluid has stronger thermal diffusivity. When we enhance the Prandtl number, then weaker thermal diffusivity appears. Such weaker thermal diffusivity creates a reduction in the temperature distribution and thermal boundary layer thickness for both fluids. Table 3 is computed to investigate the behavior of skin friction coefficient $-Re_{x}^{1/2}C_{f}$ for different values of $k_{1}^{*}.$ Tabulated values depict that the skin friction coefficient is higher for second grade fluid $\left(k_{1}^{*}<0\right)$ while opposite behavior is noticed for elastico-viscous fluid $\left(k_{1}^{*}>0\right).$ Tables 4 and 5 include the values of heat transfer rate at the surface −*θ′*(0) for various values of thermal relaxation parameter *γ* for elastico-viscous $\left(k_{1}^{*}>0\right)$ and second grade $\left(k_{1}^{*}<0\right)$ fluids respectively. Heat transfer rate at the surface −*θ′*(0) is enhanced for larger values of thermal relaxation parameter *γ* in both fluids. It is also analyzed that the values of heat transfer rate at the surface −*θ′*(0) in second grade fluid $\left(k_{1}^{*}<0\right)$ are higher when compared with the elastico-viscous fluid $\left(k_{1}^{*}>0\right).$

**Table 3:** Values of skin friction coefficient $-Re_{x}^{1/2}C_{f}$ for different values of viscoelastic parameter $k_{1}^{*}$.

$k_{1}^{*}$	−0.3	−0.2	−0.1	0.0	0.1	0.2	0.3
$-Re_{x}^{1/2}C_{f}$	1.66641	1.46059	1.23950	1.00000	0.73786	0.44721	0.11952

**Table 4:** Values of heat transfer rate at the surface −*θ*′(0) in elastico-viscous fluid when $k_{1}^{*}\;=\;0.2$ and Pr = 1.0.

*γ*	0.0	0.2	0.4	0.6	0.8
−*θ′*(0)	0.55787	0.57414	0.59165	0.61049	0.63086

**Table 5:** Values of heat transfer rate at the surface −*θ′*(0) in second grade fluid when $k_{1}^{*}\;=\;-0.2$ and Pr = 1.0.

*γ*	0.0	0.2	0.4	0.6	0.8
−*θ′*(0)	0.60023	0.61997	0.64153	0.66121	0.68288

## Conclusions

Boundary layer flows of two viscoelastic fluids over a linear stretching surface satisfying Cattaneo-Christov heat flux is analyzed. The main observations of this study are summarized below:

- Velocity profile *f′*(*η)* and momentum boundary layer thickness are reduced when we enhance the positive values of $k_{1}^{*}$ while opposite behavior is observed for the negative values of $k_{1}^{*}$.
- Both the temperature distribution *θ*(*η)* and thermal boundary layer thickness are decreased when Pr enhances.
- Increasing values of thermal relaxation parameter *γ* show a reduction in the temperature distribution *θ*(*η)* and thermal boundary layer thickness.
- Skin friction coefficient is reduced when we enhance the positive values of $k_{1}^{*}$ while opposite behavior is noticed for the negative values of $k_{1}^{*}$.
- Heat transfer rate at the surface is more for increasing values of thermal relaxation parameter *γ* for both fluids.
